# Supplementary material for: Using natural history to guide supervised machine learning for cryptic species delimitation with genetic data
Source: Front Zool. 2022 Feb 22;19:8. doi: 10.1186/s12983-022-00453-0 (PMC8862334; doi:10.1186/s12983-022-00453-0)
Supplement: Supplementary file 2 — Additional file 2: Figures S1–S12. Supplementary figures. See file for further details. [file 12983_2022_453_MOESM2_ESM.pdf]

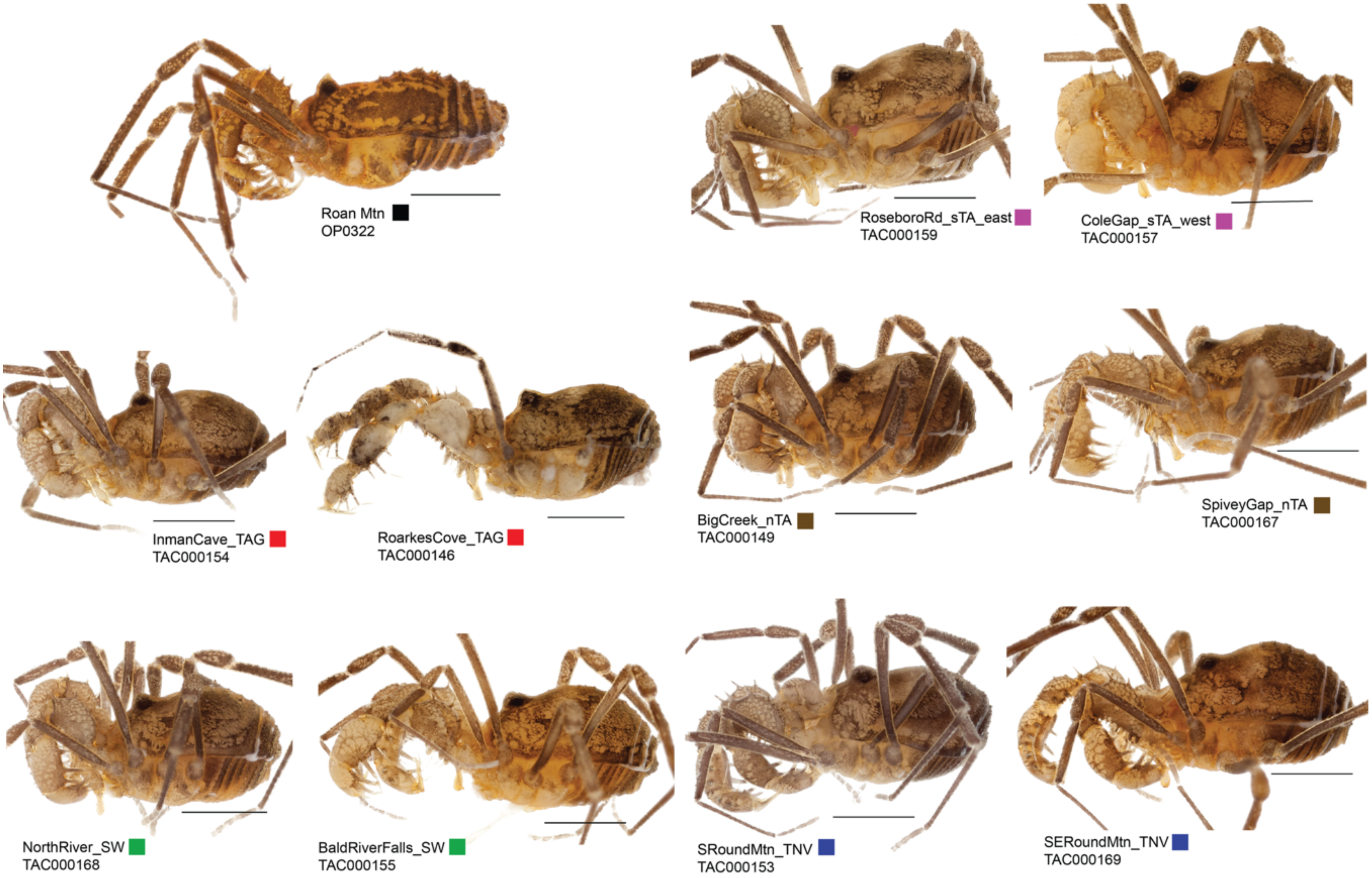

Figure S1. Habitus images of adult males. Colored squares correspond to primary phylogenomic lineages (see main text for details).

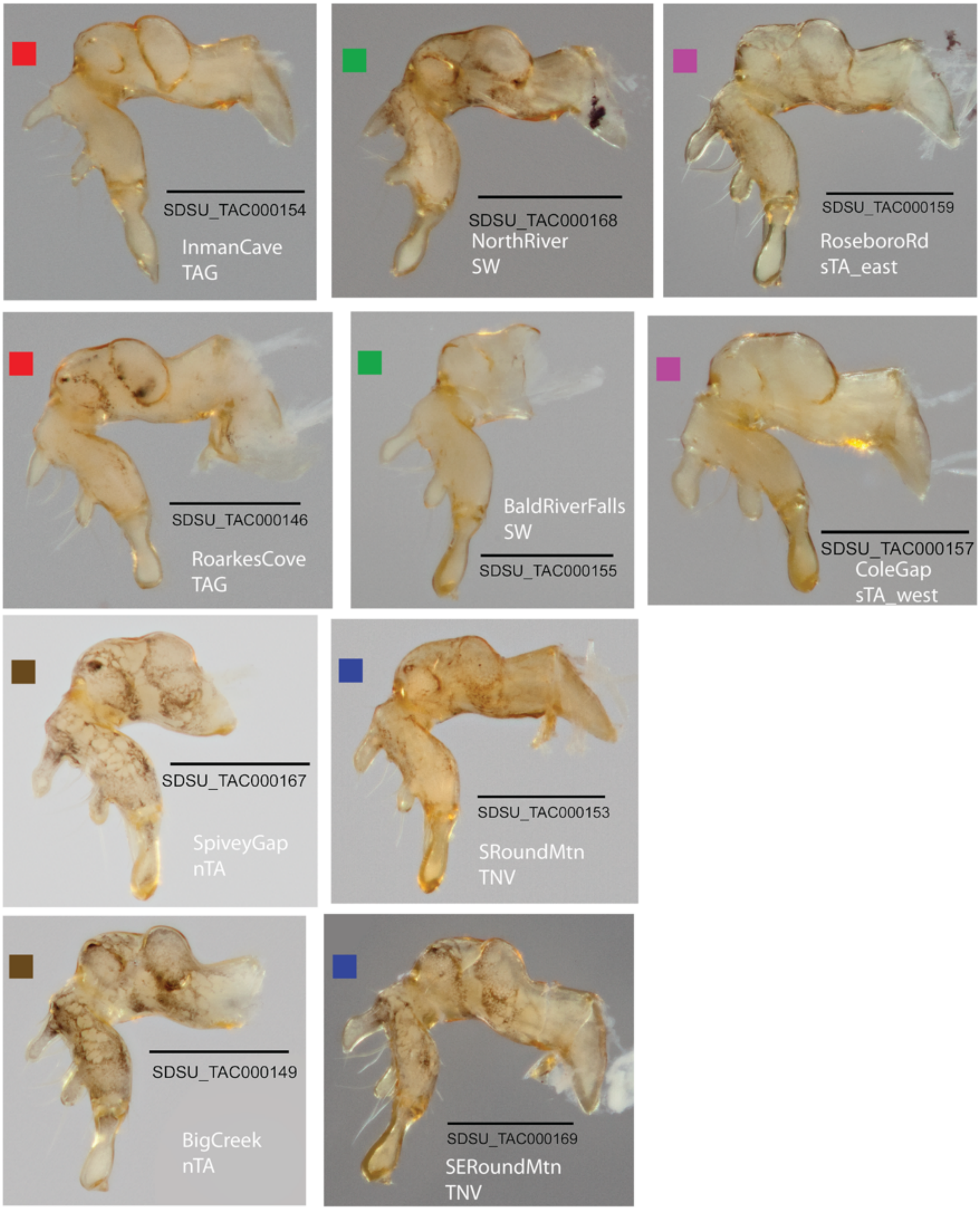

Figure S2. Digital images of adult male chelicerae. Colored squares correspond to primary phylogenomic lineages (see main text for details).

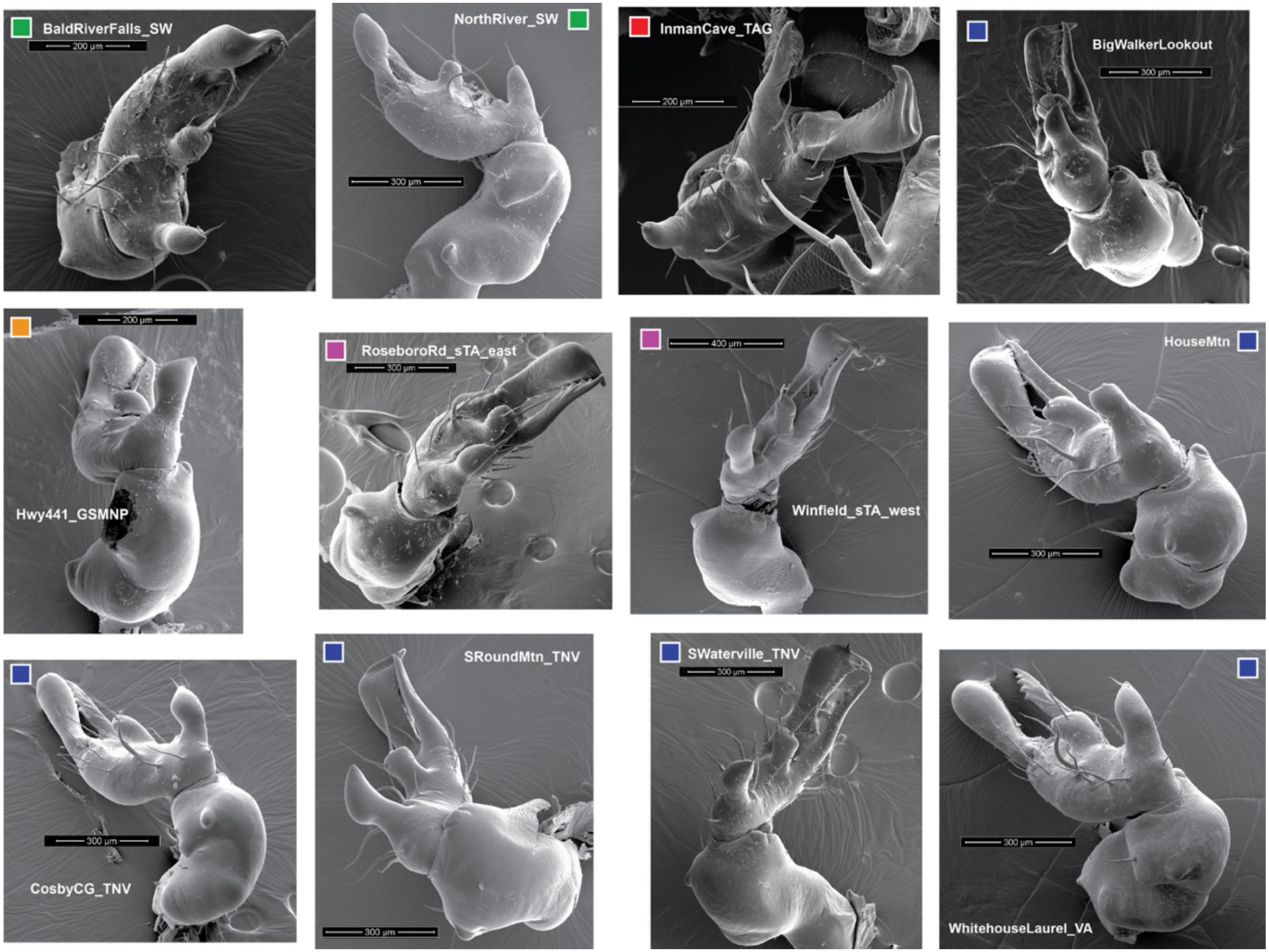

Figure S3. SEM images of adult male chelicerae. Colored squares correspond to primary phylogenomic lineages (see main text for details).

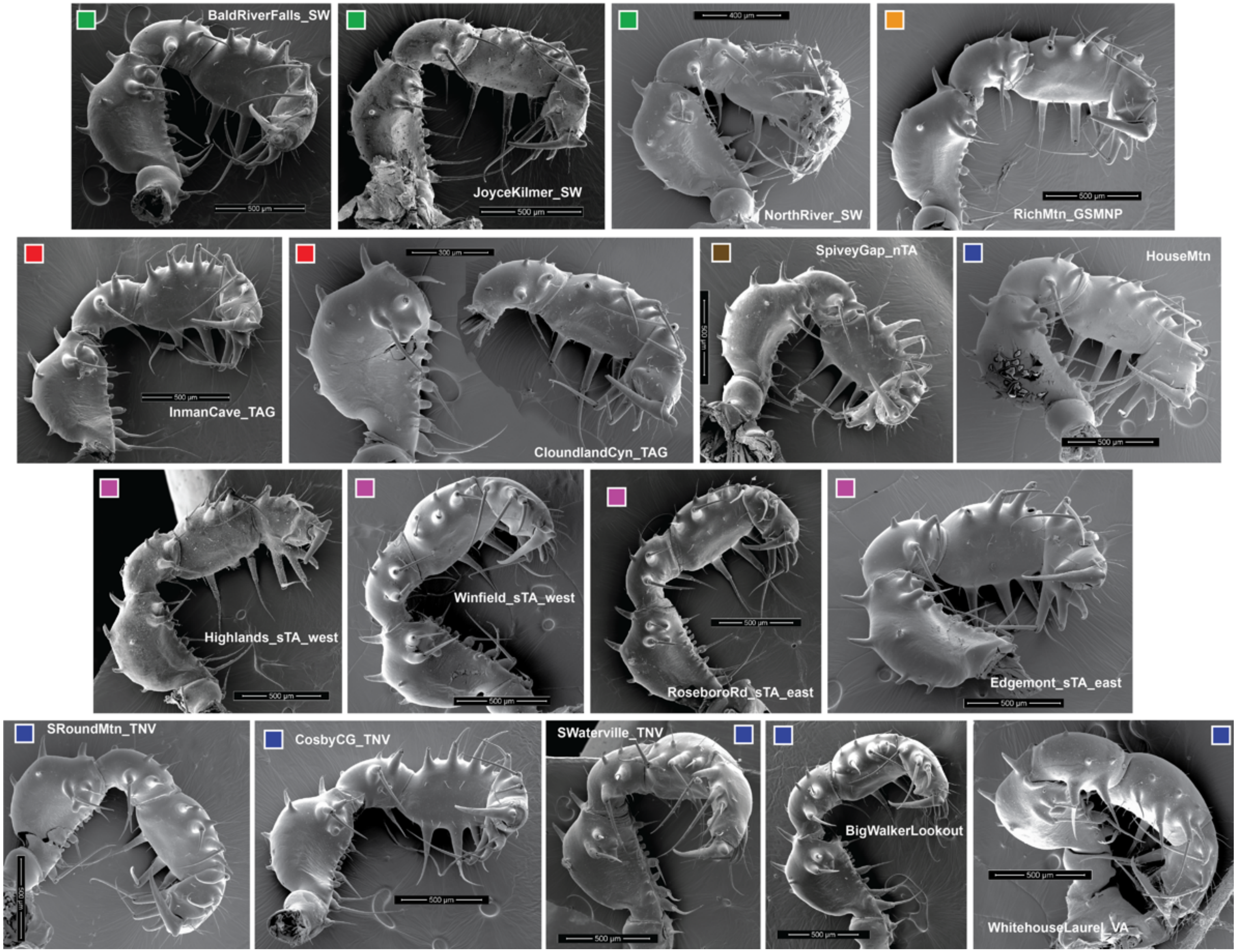

Figure S4. SEM images of adult male pedipalps. Colored squares correspond to primary phylogenomic lineages (see main text for details).

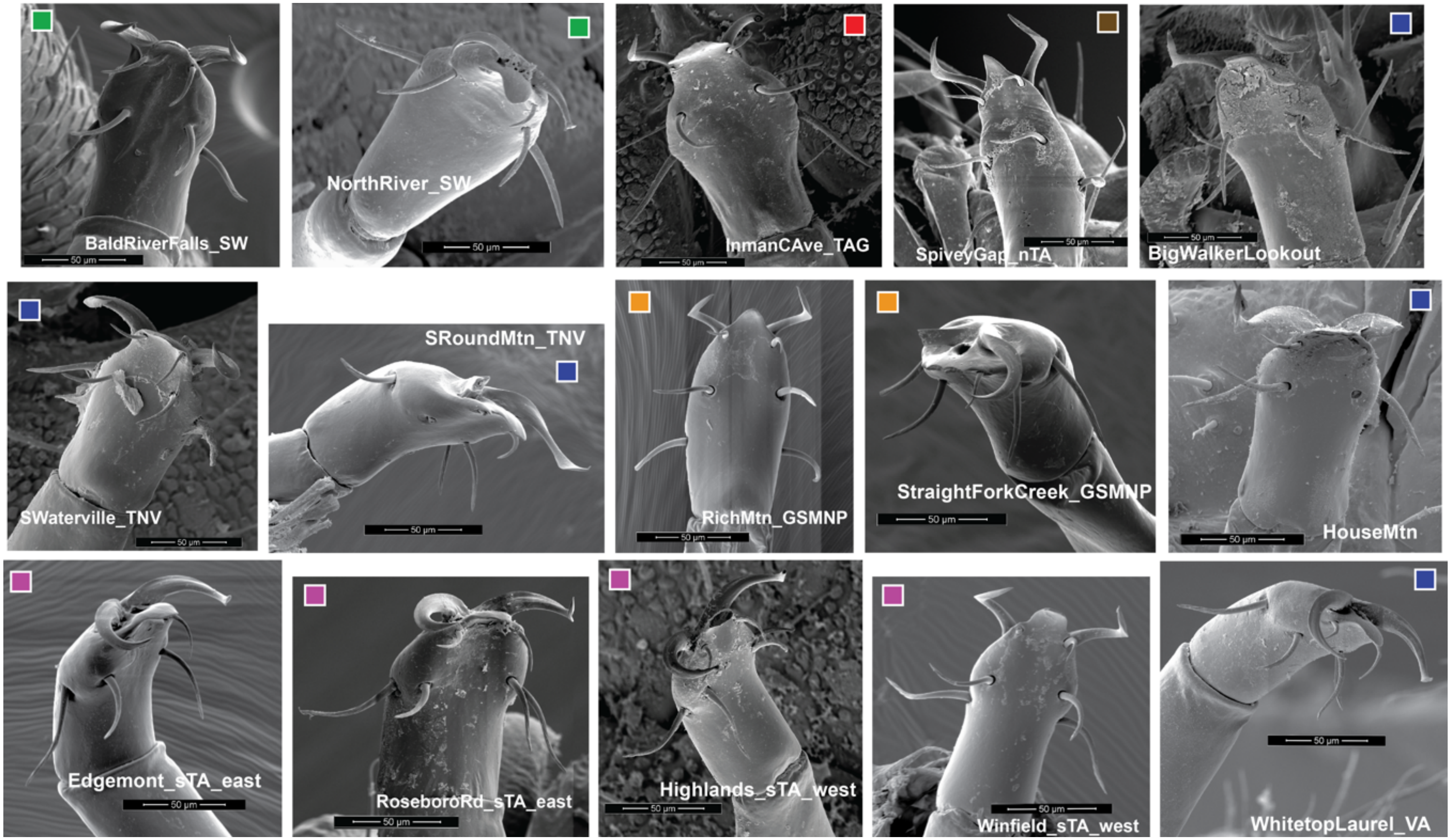

Figure S5. SEM images of adult male genitalia. Colored squares correspond to primary phylogenomic lineages (see main text for details).

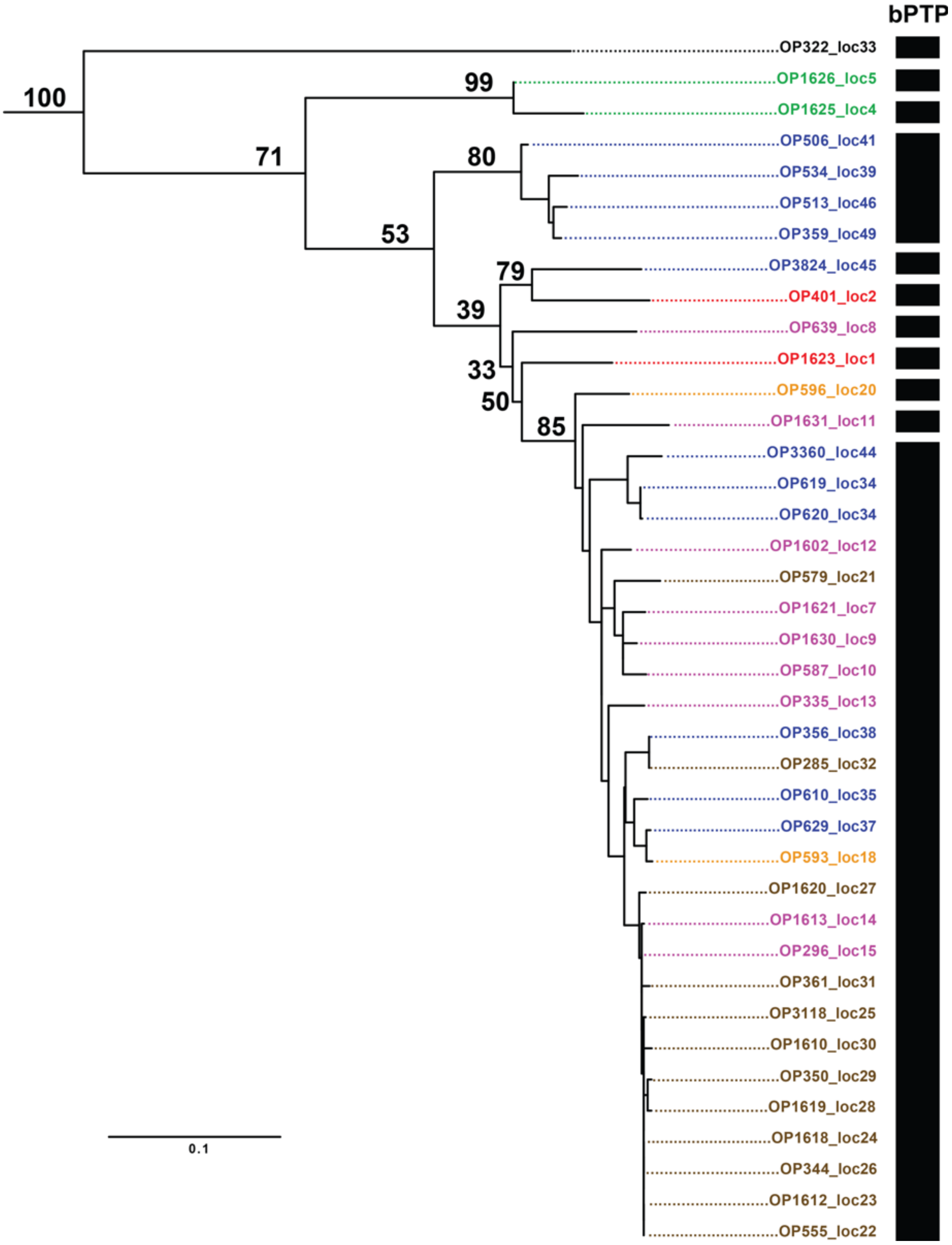

Figure S6. COI RAxML phylogeny. Bootstrap support indicated for major nodes only. Colors correspond to major phylogenomic lineages (see main text for details). Bars on the right indicate species supported by bPTP analyses.

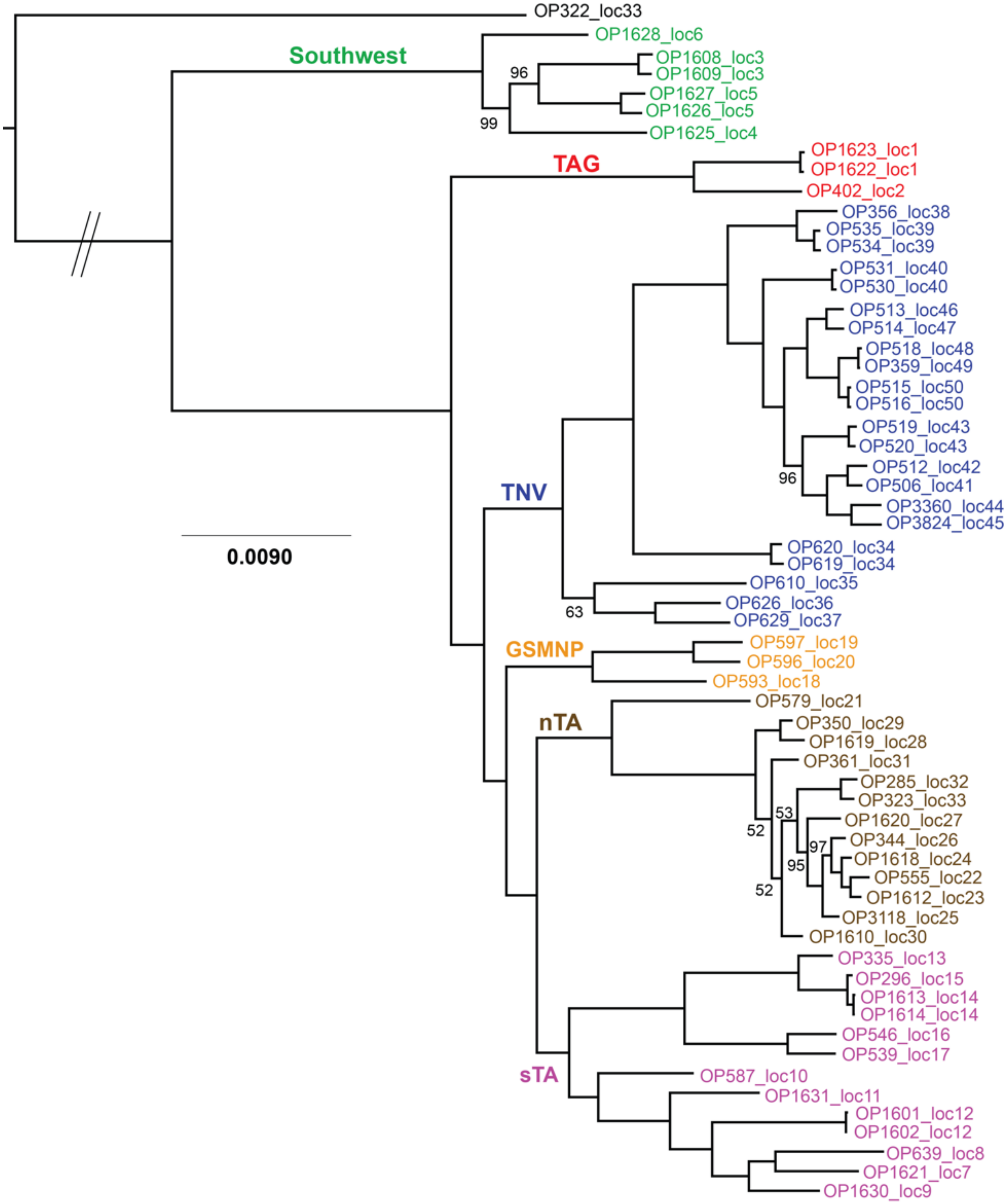

Figure S7. RAxML phylogeny of 61_31 ddRAD matrix (see Supplemental Material I for matrix details).

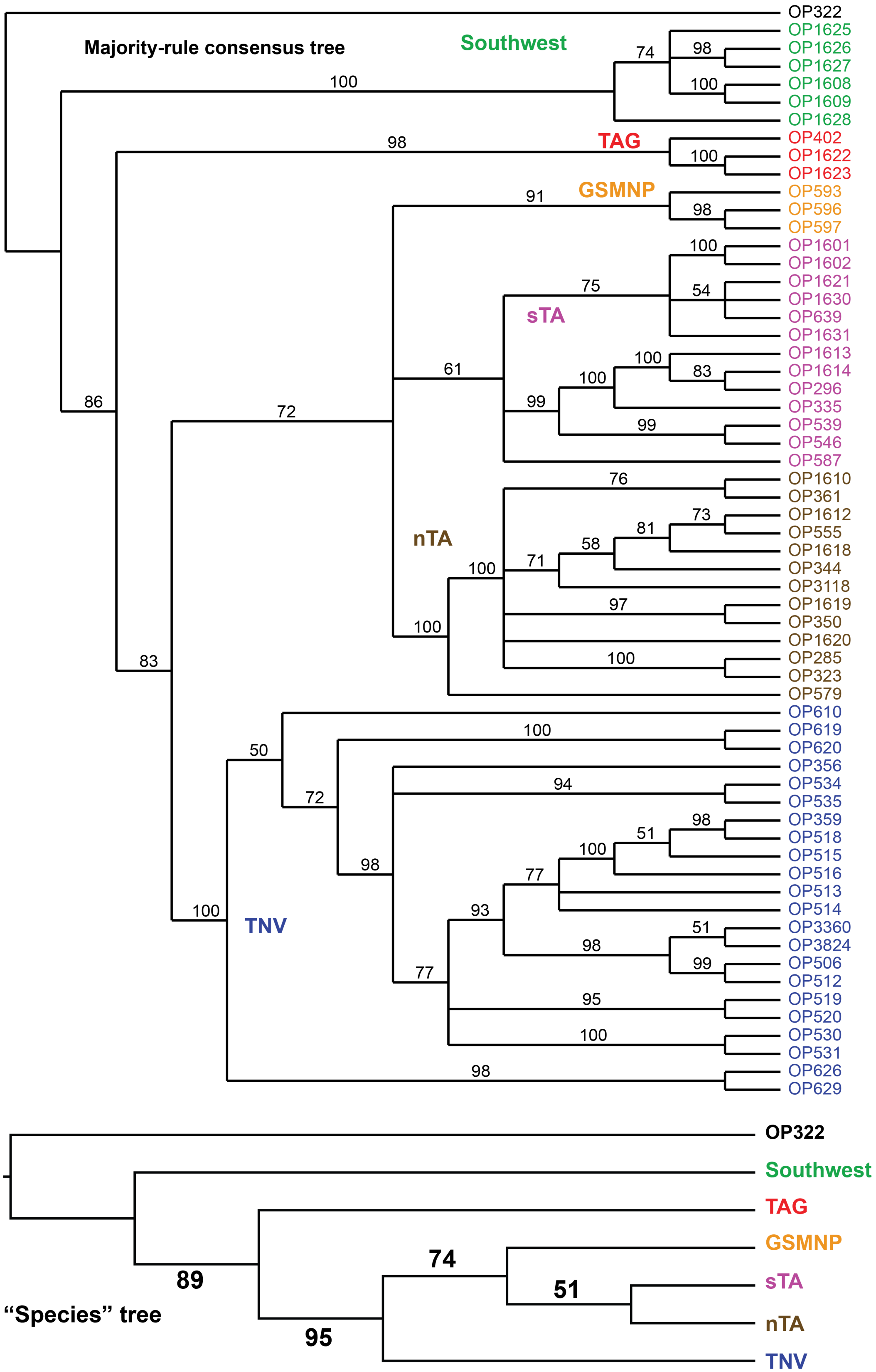

Figure S8. SVDQuartets phylogeny of ddRAD SNPs from 61_48 matrix for lineage tree (top) and species tree (bottom). See Supplemental Material I for ddRAD matrix details.

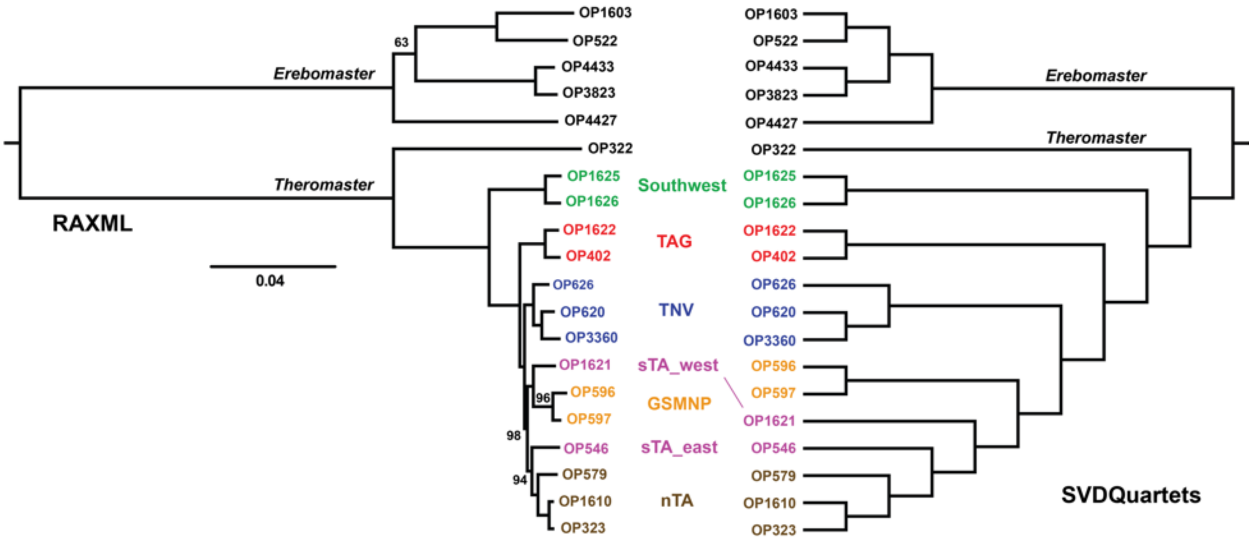

Figure S9. Phylogenomic analyses of UCE data using RAxML (left) and SVDQuartets (right) based on the 70% taxon occupancy matrix. For RAxML, all nodes have 100% bootstrap support unless indicated.

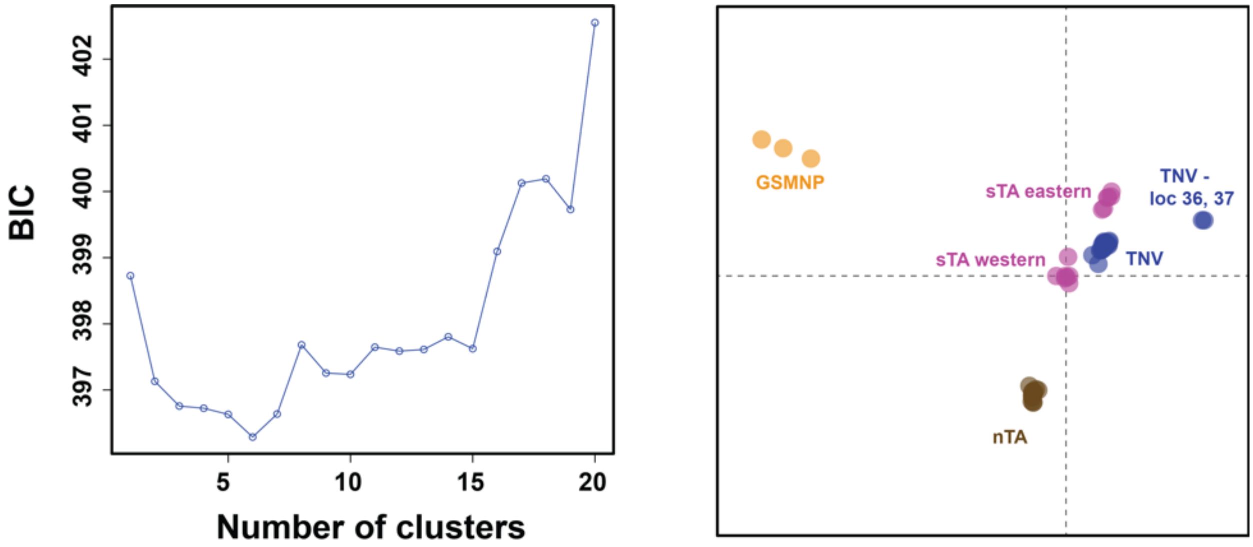

Figure S10. Results of DAPC analyses on 51_45 ddRAD SNP dataset (*see Supplemental Material 1 for matrix details).

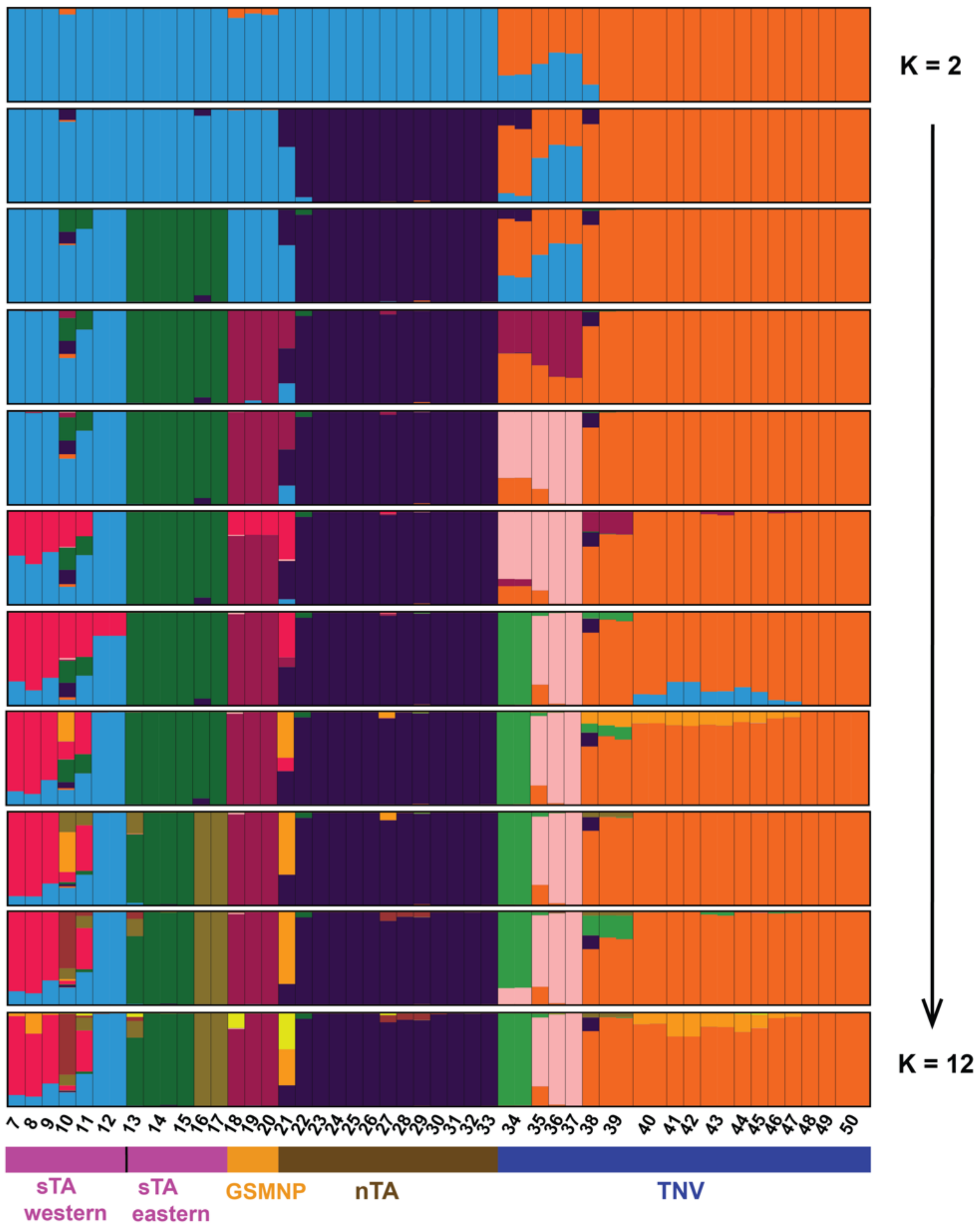

Figure S11. Results of STRUCTURE analyses on 51_45 ddRAD SNP dataset (*see Supplemental Material 1 for ddRAD matrix details).

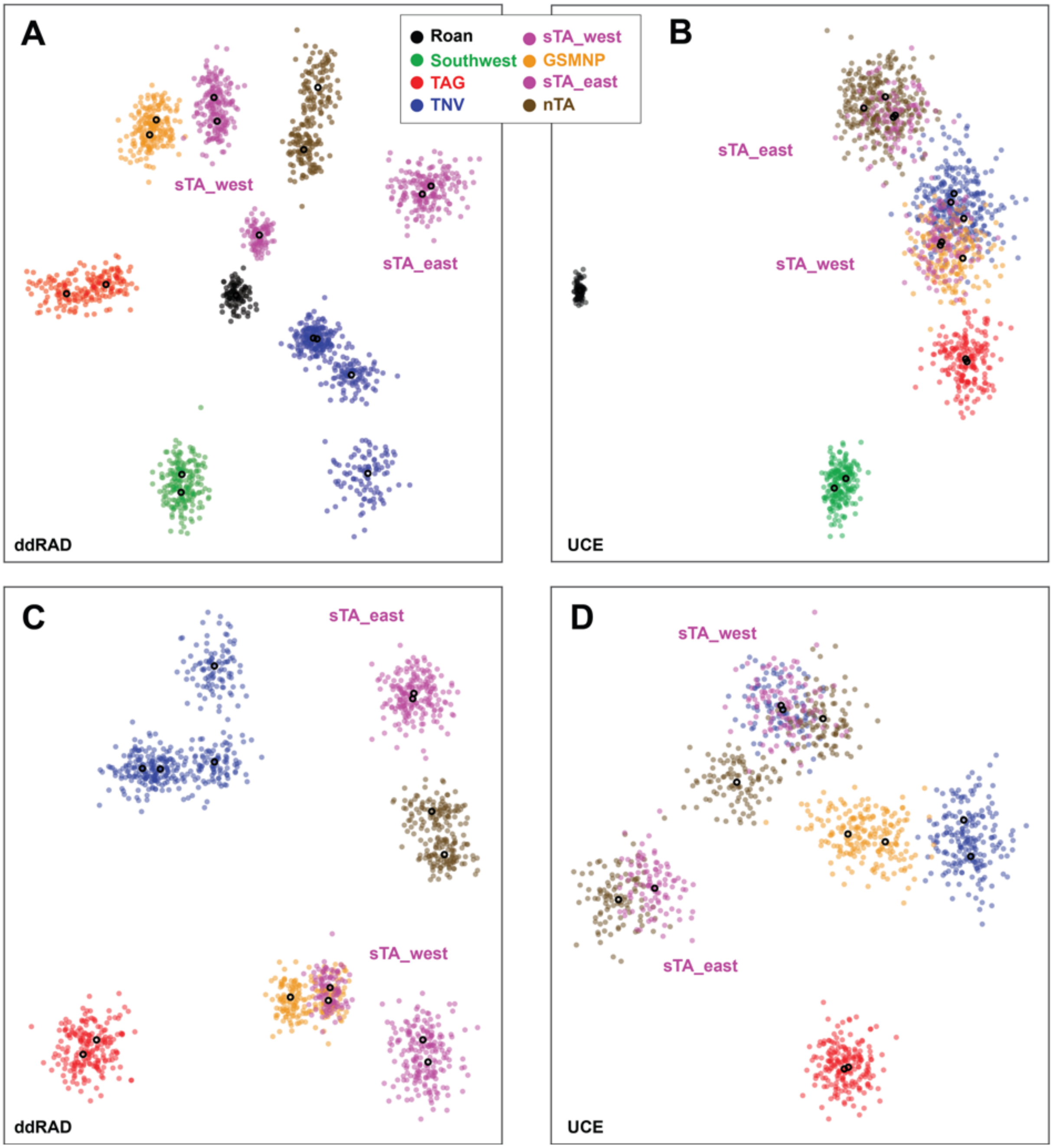

Figure S12. VAE plots for 51_45 ddRAD (A and C) and 50% taxon occupancy UCE (B and D) SNP datasets. See Supplemental Material 1 for matrix details. C and D exclude Roan Mountain and Southwest samples.
